# Creating Novel Standards for Datapoints on an Elective Orthopaedic Theatre List Document

**DOI:** 10.5704/MOJ.2407.002

**Published:** 2024-07

**Authors:** M Raad, S Virani, S Vinay, P Housden

**Affiliations:** Department of Trauma and Orthopaedics, William Harvey Hospital, Ashford, United Kingdom

**Keywords:** orthopaedic theatres, new guidelines, efficiency, theatre team, theatre lists

## Abstract

**Introduction:**

Orthopaedic theatre lists are an important tool which must convey essential information to all staff to run an effective and safe theatre list. However, there are no set standards or guidelines on the components of an Orthopaedic theatre list. The objective of this study is to formulate guidelines for elective Orthopaedic theatre lists which improve efficiency and reduce errors.

**Materials and Methods:**

We looked at 326 elective Orthopaedic theatre lists from October to November 2018. Various factors such as: theatre and patient demographics, surgical team, type of anaesthesia, Surgery, acronyms and finally extra information such as allergies. Additionally, a survey was distributed to a variety of theatre staff to understand their requirements from a theatre list. Thereafter, we created a proforma for waiting list coordinators. Subsequently, we re-audited six more weeks of theatre lists (255) from November to December 2019.

**Results:**

The orthopaedic consultant in charge was noted for 100% of patients compared to 85% previously. There was an improvement in documenting the required anaesthesia such as noting 14.5% required spinal compared to 0.3% previously. Prosthesis/equipment was mentioned for 34% of patients compared to 23%. Fluoroscopy was noted as being required for 25% of patients compared to 11%.

**Conclusion:**

We believe standards should be in place in order for us to follow to ensure we carry out safe and efficient Orthopaedic theatre lists, and these standards should entail the parameters we have audited. The ‘William Harvey theatre list standard’ should be used as a gold standard for all elective Orthopaedic theatre lists.

## Introduction

Surgical departments are increasingly put under pressure to improve services, cut waiting lists, increase efficiency, and save money^1^. Orthopaedic theatre lists are an important tool which must convey essential information to all staff to run an effective and safe theatre list. There are currently no standards or guidelines on the components of an Orthopaedic theatre list. The objective of this study is to identify the information on a theatre list that is most valued by those who use it and formulate guidelines to improve efficiency and reduce errors.

Theatre efficiency has gained increasing attention though the Productive Operating Theatre (TPOT) initiative from the NHS Institute for Innovation and Improvement^2^. However, literature specifically mentioning what should be on an Orthopaedic theatre list is limited. Running a theatre is expensive and so it is essential to maximise efficiency (NHS III 2009)^2^.

The operating room is home to both lifesaving, and quality of life saving intervention. It is also one of the most expensive areas to maintain for an NHS trust. Therefore, effective cohesion, utilisation, and efficiency of: the theatre space, pre-operative planning, and theatre staff are of paramount importance when considering improvements to that system^1^. Delays and cancellations in theatre may lead to an increased length of hospital stay, complications and patient complaints. Subsequently, this can all lead to increase costs incurred by the trust.

## Materials and Methods

**Fig. 1:**
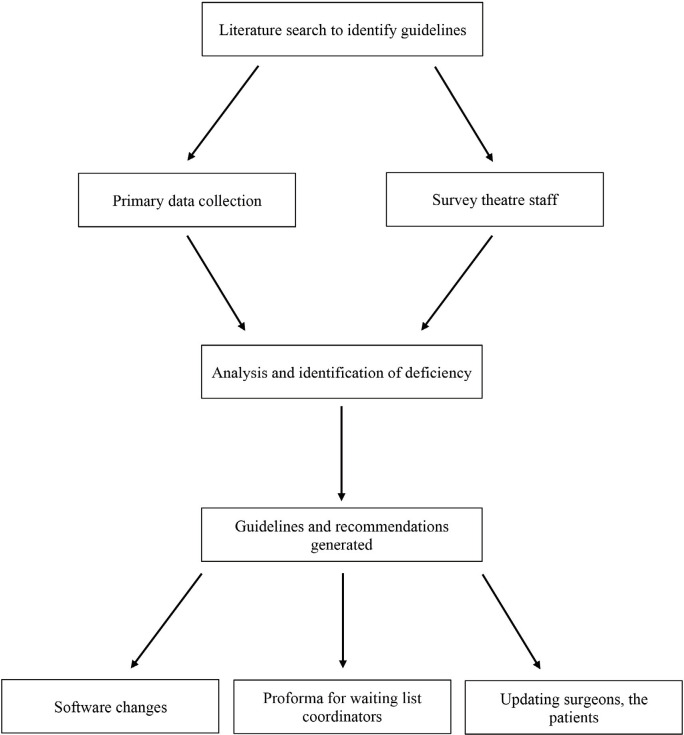
Flowchart for methodology.

We devised a structured approach to enable us to carry out this quality improvement project (Fig. 1). A literature search was carried out initially in order to find a standard used when compiling an elective Orthopaedic theatre list. Despite a thorough search we were unable to find a standard.

We performed a retrospective study using data gathered from Theatreman [Trisoft Ltd Nottingham, U.K], the operating theatre database at our institution to critically assess and compile the information conveyed by the board pinned theatre list. In the first round of our study, we collected six weeks of data from 1st October 2018 to 11th November 2018. This consisted of 115 operating lists and 326 cases which included all elective Orthopaedic theatre lists excluding spine surgery and trauma cases. The lists were assessed for presence and detailing of several datapoints like theatre and patient demographics, Surgical team (Consultant in charge, operating Surgeon, first assistant, lead Anaesthetist), type of anaesthesia (general anaesthesia (GA), local anaesthesia (LA), regional, sedation), Surgery (side, operation, prothesis/equipment, cemented/uncemented, fluoroscopy or medical representative requirement), use of acronyms, critical extra information such as allergies, infection, disabilities, comorbidities, high body mass index (BMI) and whether a post-operative high dependency unit (HDU) or intensive care bed was required. The data collected was consolidated in an Excel sheet [Microsoft, Corp. Redmond, WA].

**Fig. 2:**
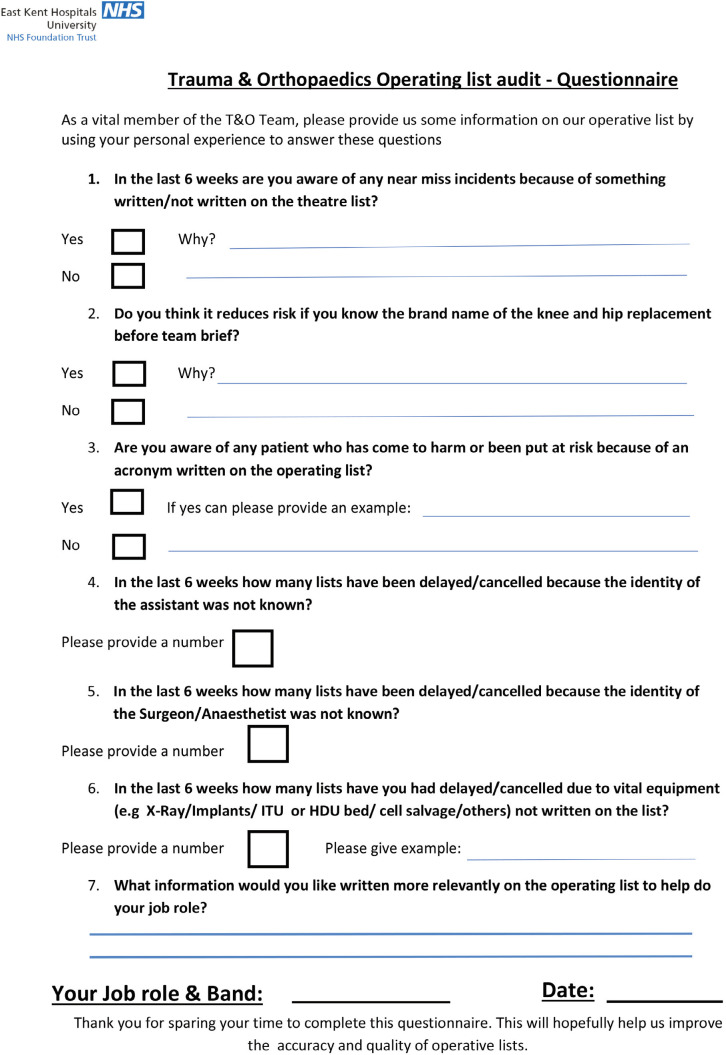
Trauma and orthopaedics operating list questionnaire.

Simultaneously, we distributed 40 questionnaires to a variety of Orthopaedic theatre staff over a one-week period (Fig. 2). From these questionnaires 38 were fully completed. The aim of the questionnaire was to assess the opinions on deficiencies in theatre list from an all-round perspective. Of these questionnaires, six were filled out by a Consultant Anaesthetist, five by an Orthopaedic theatre sister, five by surgical care practitioners (SCP), four by operating department practitioners (ODP), six by scrub nurses, eight by theatre support workers (TSW), one by an assistant theatre practitioner (ATP) and three by a band two nurse.

On the basis of deficiencies identified from our objective data analysis and inputs from the staff questionnaire, two observers put together the optimal data points that must be displayed on an orthopaedic theatre list. Further, a set of guidelines were created for individuals who populate a theatre list (consultants, trainees and waiting list coordinators).

The next step was carrying out an intervention to ensure uptake of these newly created standards. The data and recommendations were discussed in the audit meeting and approval was gained for their implementation. The information-technology (IT) team of the hospital were then involved to make a few minor changes in the input form for theatre list to ensure compliance. Further, waiting list coordinators and trainees were then educated about this change and concerns or glitches if any were addressed on a regular basis.

To assess the effectiveness of the intervention, a re-audit was carried out from 1st November 2019 to 15th December 2019, the same inclusion and exclusion criteria as before. This data was consolidated and then compared with the previous data to assess for objective improvement in practice. This was followed by a second survey of a variety of staff to assess any objective or subjective improvement in practice.

## Results

In the first round of our study, we collected six weeks of data from 1st October 2018 to 11th November 2018, this consisted of 115 operating lists and 326 cases. Of these, 112 were elective knee cases of which 52 were total knee replacements. Sixty-nine cases were elective hip cases of which 56 were total hip replacements. Forty-five were elective shoulder and elbow cases and 100 were elective hand and foot cases. The second round of data was carried out from 1st November 2019 to 15th December 2019, consisting of 100 operating lists and 255 cases. Of these, 73 were elective knee cases of which 53 were total knee replacements, 78 were elective hip cases of which 56 were total hip replacements, 44 were elective shoulder and elbow cases and 60 were elective hand and foot cases.

**Fig. 3:**
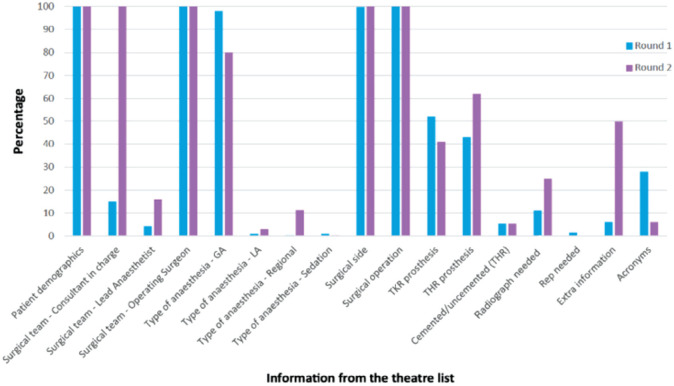
Bar chart comparing both rounds of data collection.

Whilst comparing our results we found that 100% of theatre lists had all necessary theatre and patient demographics in both rounds of data. In regard to the surgical team: 15% did not mention the lead Orthopaedic Consultant however in the second round of data 100% of lists had a lead Orthopaedic Consultant mentioned. All lists mentioned an operating surgeon in both rounds of data, 4.3% did not mention who the Lead Anaesthetist was in the first round of data compared to 16% of lists without a lead Anaesthetist in the second round of data (Fig. 3).

With regard to the type of anaesthesia, 98% of cases were listed as GA, although 55% of the cases were hip or knee surgeries which are commonly done under regional anaesthesia. Only three patients were listed as LA, one patient as regional and three patients as sedation. In the second round of data, we found 80% of cases were listed as GA, although 44% of the cases were hip or knee surgeries. Ten patients were listed as LA, 37 patients as regional and one patient as sedation.

In regard to the surgery: the first round of data showed the side was not mentioned for one patient, however 100% of cases had a side in the second round of data. In both rounds of data 100% of cases had the procedure name mentioned. However, 76.8% of patients did not have any detail in regard to the prosthesis or equipment described, this decreased to 43% in the second round of data. We looked at hip and knee arthroplasty surgery separately, 52% of total knee arthroplasty cases mentioned the prosthesis required and 41% of total hip arthroplasty cases had specified on prosthesis required. In the second collection of data, 43% of total knee arthroplasty cases mentioned the prosthesis required and 62% of total hip arthroplasty cases had specified on prosthesis required. In both rounds of data only 5.3% of hip arthroplasty cases specified if cement was required, although 21% of hip replacements were cemented. Whilst looking at whether fluoroscopy or Rep requirement was mentioned, in the first round of data, 26 cases required fluoroscopy and 11% of these mentioned if fluoroscopy was required. In the second set of data, 24 cases required fluoroscopy and 25% of these had mentioned if fluoroscopy was required (p<0.5). Whether a Rep was required was mentioned for 1.5% of cases in the first round of data but it was not mentioned for any case in the second round of data. Regarding acronyms, 28% of cases had mentioned a type of acronym in the first round of data compared to 6% in the second round of data (p<0.5). Finally, looking at extra information mentioned only 6% of cases listed necessary extra information required in regard to the patient. This increased to 49.8% in the second round of data.

From the completed questionnaires we received a variety of feedback from Orthopaedic theatre staff for example: a band six scrub nurse mentioned radiograph was not identified on elective list which delays trauma lists, another band six mentioned kit hadn’t been ordered and the need to mention the type of implant required and a band five mentioned there was the wrong side on the list. A senior ODP mentioned no intensive care bed was available for some cases, an

Orthopaedic scrub nurse mentioned kit was lacking and had to be ordered from another site and a Consultant Anaesthetist remarked that it would be useful to have diabetes status and BMI.

After our first round of data collection, we developed a proforma for the waiting list coordinators which entailed essential information required for elective Orthopaedic theatre lists (Table I).

**Table I:** Table for the waiting list coordinators with required essential information.

Patient Demographics	Surgical Team	Type of anesthesia	Surgery	Extra information
Name	Consultant in charge	GA	Side	Allergies
Hospital Number	Lead Anaesthetist	LA	Surgical operation	Significant comorbidities e.g Diabetes Mellitus
Date of birth	Operating Surgeon	Regional	Prosthesis/equipment needed	Disabilities
Age	First assistant	Sedation	Cemented/uncemented (THR)	High BMI
Gender			Radiograph needed Rep needed	

We asked for them to ensure all information was placed on the booking form and if not mentioned in the booking to go back to the Surgeon booking the patient and ask them for any missing information. The Trust IT team was involved in this discussion to make the proforma more compliant with these standards.

Following the results of our first round of data collection and after putting our intervention into place we did a re-audit. Results showed there was a 15% improvement in mentioning the Operating Surgeon. There was a 34% rise in the number of patients listed for spinal anaesthesia and a 7% rise in listed patients for local anaesthesia appropriately, therefore leading to a 18% decrease in listing patients for general anaesthesia (p<0.5). In the re-audit 100% of operations mentioned the side and there was an 11% improvement in mentioning which prosthesis or equipment was required. Furthermore, there was an improvement in stating if radiograph was required of 14%. Lastly, there was a 49.2% increase in adding critical extra information for patients.

It is also important to note that during the time of our re-audit, there were no delays in theatre due to lack of kit or radiograph requirements. It was clear beforehand, for every list who the lead Consultant Anaesthetist and operating Surgeon would be. Based on our results and due to the lack of standards, we have designed our own standard based on the parameters we have been analysing (Table II).

**Table II:** Table of our own novel standards for a trauma and orthopaedic theatre list.

Patient Demographics	Surgical Team	Type of anesthesia	Surgery	Radiograph required	Rep needed	Extra information
Name	Consultant in charge	GA	Side	Fluoroscopy		Allergies
Hospital Number	Lead Anaesthetist	LA	Operation	Mini C-arm		Diabetes Mellites
Date of birth	Operation Surgeon	Regional	Prosthesis/equipment needed			Significant comorbidities
Age		Sedation	Cemented/uncemented (hip arthroplasty)			Disabilities
Gender			Radiograph needed			Infection
			Rep needed			High BMI

## Discussion

Our study shows some interesting results with regards to improvement in the information conveyed by theatre lists. We know that information like type of prosthesis in hip and knee replacements, probable bearing surfaces in hip replacements, type of implants/kit for other elective orthopaedic surgery would help the surgical team be better prepared in advance for the theatre list. This would likely ensure faster turnover time between cases, efficient usage of theatre time and fewer cancellations due to lack of equipment, capacity or manpower. Standardising and reducing supplies and durable instruments have benefits inside and outside the operating room by reducing operative costs, setup, counting and turnover times^3^. Farrokhi *et al* applied lean methodology to reduce surgical trays for minimally invasive spine surgery by 70% (197 tools to 58) and decrease operative time by 7 min^4^. Instrument reduction is another way for surgeons to get immediate efficiency improvements and nurses will need less time to prepare a room^5^. Cerfolio *et al* identified a workflow issue with the circulator and eliminated unnecessary travel time to retrieve supplies by stocking the case cart for the day with the required supplies^6^.

Unexpected cancellations of elective surgical procedures can result in significant potential losses for hospital systems. In 2007, hospitals in the UK lost almost $88 million for cancelled operations^7^. A large proportion of the cancellations are often preventable, in some institutions accounting for as much as 50-70% of the cancellations^8^. Hospital-related factors include missing or failed equipment, prioritising emergent surgeries, or lack of hospital beds. Surgeon/staff-related factors include unavailability of required essential staff, Surgeons, or Anaesthesiologists^9^. Sultan *et al* looked at 41 cases, only 54% of theatre time was utilised for operating, the anaesthetic time was 12.0%, and 9.3% of theatre time was used for positioning and draping. Delays in starting the list and turnover time accounted for the remaining 2510. Kaddoum *et al* analysed that 71.96% (187 cases) of elective surgeries were potentially avoidable cancellations and lack of financial clearance, incomplete medical evaluation, patient not showing up for surgery, and theatre time behind schedule accounted for almost 80% of the causes^11^. The requirement of the instruments necessary for scheduled surgical list should be discussed a day prior to planned operating list and arranged^12^. Additional components like fluoroscopy would help the theatre co-ordinator plan for allocation of resources like the c-arm and radiographer which at times could be limited in availability. A short note about a significant health condition or allergy would help not only the Anaesthetist but also the theatre managers to make sure an HDU or intensive care bed is available for major surgeries in high-risk patients. More efficient use of elective orthopaedic theatre sessions is possible and could be achieved if more detailed preparation was undertaken by the anaesthetic, theatre and surgical staff concerned. If a consultant surgeon is present the list is likely to proceed with fewer delays^13^. Chamisa found that of a total of 5,786 operations, 5.6% were cancelled or postponed. Lack or failure of instruments and patient cancellation constituted 2.8% and 1.8% of the cancellations, respectively^14^.

Standards and guidelines are a useful way to improve efficiency as they ensure uniformity across teams, departments, and hospitals. The application of these standards increased the relay of important information like requirement of special kit, need for fluoroscopy, need for a medical representative. It also forewarned the anaesthetist and bed managers of any significant medical condition and potential need of HDU/ITU support post-operatively. Finally, these standards reduced the use of non-approved acronyms thereby reducing confusion and ensuring all staff were on the same page. The subjective effect of this change was appreciated in the follow-up survey which included stakeholders like anaesthetists, scrub nurses, ODPs and surgeons. We hope that the standards created would help improve theatre efficiencies by providing comprehensive and relevant information to the theatre teams. It is apparent that the increased efficiency would allow more operations to be scheduled per day and thus result in shortened waiting lists reducing patient discomfort^15^. We hope to assess the objective impact of these interventions on improving theatre efficiencies in the future. We called this set of standards as the ‘William Harvey theatre list standard’ after the hospital where it was first designed and implemented. Further, the acceptability of these standards for orthopaedic trauma surgery needs to be determined as well. The next step would be involving regional and national policy makers and steering groups to assess the impact when these standards are applied across multiple sites. We believe these guidelines would be a first step in the process of firming up definitive standards for an ideal theatre list document.

Limitations to our study include getting approval for funding to make changes to our Theatreman system to add mandatory fields, therefore all interventions could not be done due to cost. This is a single centre study. Also, there is subjective correlation between efficiency improvement and compliance with suggested standards.

Finally, we have identified a literature gap in regard to improving efficiency in Orthopaedic elective theatres in particular in hip and knee arthroplasty which presents the need for further development in this area.

In the limitations, the aim of our study was to formulate effective guidelines for elective Orthopaedic theatre lists. We limited our study to elective procedures in hip, knee and foot and ankle surgery to limit the number of variables that had to be audited in the given time period. Furthermore, at the time of our study we did not have many regular elective spines lists so we did not include these. However, in the future we will bring this new proforma forward into the elective spine lists and ensure the proforma is adaptable for all elective orthopaedic cases. Furthermore, we did not include trauma lists as the William Harvey hospital is a busy trauma unit. Sometimes an emergency can come in which takes priority ahead of other planned trauma cases and in general patients have more comorbidities and may take longer to optimise on the ward or in the anaesthetic room. In some cases, it is unclear which comorbidities a patient has and unlike elective patients they do not have a pre-operative assessment, majority of the time they are seen by the Anaesthetist on the day of the surgery. Therefore, it is expected cancellations or delays can happen. However, a similar proforma for regular trauma and Orthopaedic lists should be in place to ensure a smoother running list.

## Conclusion

Availability of vital information before the day of the surgery ensures the surgical, anaesthetic and nursing team are better prepared for the operating list. This in turn, leads to increase in patient safety, prevention of near misses and never events. We believe using the ‘William Harvey theatre list standard’ is the beginning to create a gold standard for all elective Orthopaedic theatre lists.
